# Inhibitory Effects of Caffeic Acid Phenethyl Ester Derivatives on Replication of Hepatitis C Virus

**DOI:** 10.1371/journal.pone.0082299

**Published:** 2013-12-17

**Authors:** Hui Shen, Atsuya Yamashita, Masamichi Nakakoshi, Hiromasa Yokoe, Masashi Sudo, Hirotake Kasai, Tomohisa Tanaka, Yuusuke Fujimoto, Masanori Ikeda, Nobuyuki Kato, Naoya Sakamoto, Hiroko Shindo, Shinya Maekawa, Nobuyuki Enomoto, Masayoshi Tsubuki, Kohji Moriishi

**Affiliations:** 1 Department of Microbiology, Division of Medicine, Graduate School of Medicine and Engineering, University of Yamanashi, Yamanashi, Japan; 2 Faculty of Pharmaceutical Sciences, Toho University, Chiba, Japan; 3 Institute of Medical Chemistry, Hoshi University, Tokyo, Japan; 4 Department of Tumor Virology, Okayama University Graduate School of Medicine, Dentistry, and Pharmaceutical Sciences, Okayama, Japan; 5 Department of Gastroenterology and Hepatology, Hokkaido University Graduate School of Medicine, Sapporo, Japan; 6 First Department of Internal Medicine, Faculty of Medicine, University of Yamanashi, Yamanashi, Japan; Kobe University, Japan

## Abstract

Caffeic acid phenethyl ester (CAPE) has been reported as a multifunctional compound. In this report, we tested the effect of CAPE and its derivatives on hepatitis C virus (HCV) replication in order to develop an effective anti-HCV compound. CAPE and CAPE derivatives exhibited anti-HCV activity against an HCV replicon cell line of genotype 1b with EC_50_ values in a range from 1.0 to 109.6 µM. Analyses of chemical structure and antiviral activity suggested that the length of the n-alkyl side chain and catechol moiety are responsible for the anti-HCV activity of these compounds. Caffeic acid n-octyl ester exhibited the highest anti-HCV activity among the tested derivatives with an EC_50_ value of 1.0 µM and an SI value of 63.1 by using the replicon cell line derived from genotype 1b strain Con1. Treatment with caffeic acid n-octyl ester inhibited HCV replication of genotype 2a at a similar level to that of genotype 1b irrespectively of interferon signaling. Caffeic acid n-octyl ester could synergistically enhance the anti-HCV activities of interferon-alpha 2b, daclatasvir, and VX-222, but neither telaprevir nor danoprevir. These results suggest that caffeic acid n-octyl ester is a potential candidate for novel anti-HCV chemotherapy drugs.

## Introduction

Hepatitis C virus (HCV) is well known as a major causative agent of chronic liver disease including cirrhosis and hepatocellular carcinoma and is thought to persistently infect 170 million patients worldwide [1]. HCV belongs to the genus *Hepacivirus* of the family *Flaviviridae* and possesses a viral genome that is characterized by a single positive strand RNA with a nucleotide length of 9.6 kb [2]. The single polypeptide coded by the genome is composed of 3,000 amino acids and is cleaved by host and viral proteases, resulting in 10 proteins, which are classified into structural and nonstructural proteins [3]. The viral genome is transcribed by a replication complex consisting of NS3 to NS5B and host factors [4]. NS3 forms a complex with NS4A and becomes a fully active form to cleave the C-terminal parts of the nonstructural proteins. The advanced NS3/4A protease inhibitors, telaprevir and boceprevir, have been employed in the treatment of chronic hepatitis C patients infected with genotype 1 [5]. Sustained virologic response (SVR) was reportedly 80% in patients infected with genotype 1 following triple combination therapy with pegylated interferon, ribavirin, and telaprevir [6], although the therapy exhibits side effects including rash, severe cutaneous eruption, influenza-like symptoms, cytopenias, depression, and anemia [7]. In addition, there is the possibility of the emergence of drug-resistant viruses following treatment with those anti-HCV drugs [8] Thus, further study is required for development of safer and more effective anti-HCV compounds.

**Figure 1:**
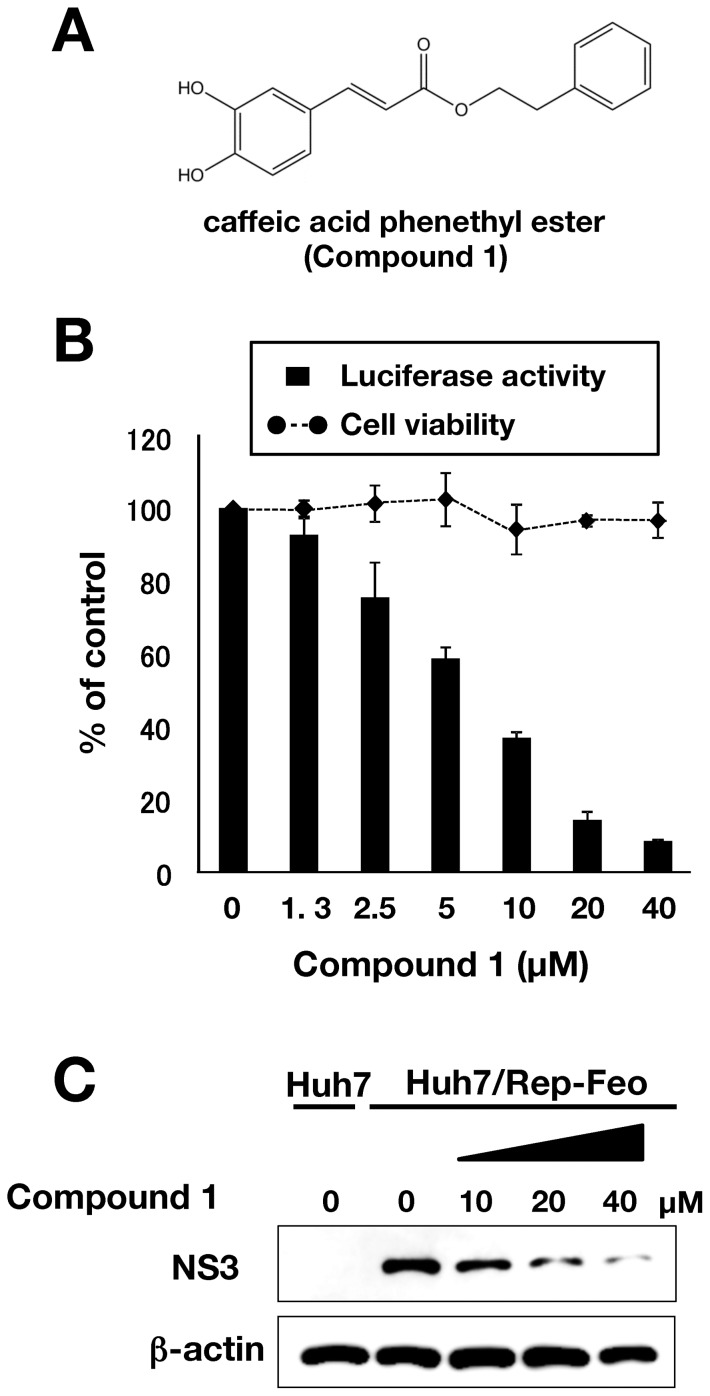
Effect of CAPE on viral replication in the replicon cell line of genotype 1b. (A) Molecular structure of CAPE. (B) Huh7/Rep-Feo cells were incubated for 72 h in a medium containing various concentrations of CAPE. Luciferase and cytotoxicity assays were carried out by the method described in Materials and Methods. Error bars indicate standard deviation. The data represent results from three independent experiments. (C) Protein extract was prepared from Huh7/Rep-Feo cells treated for 72 h with the indicated concentration of CAPE and it was then was subjected to Western blotting using antibodies to NS3 and beta-actin.

Several recent reports indicate that silbinin [9], epigallocatechin-3-gallate [10], curcumin [11], quercetin [12] and proanthocyanidins [13], which all originate from natural sources, have exhibited inhibitory activity against HCV replication in cultured cells. Caffeic acid phenethyl ester (CAPE) is an active component included in propolis prepared from honeybee hives, and has a similar structure to flavonoids (Fig. 1A). CAPE has multi-functional properties containing anti-inflammatory [14], antiviral [15], anticarcinogenic [16], and immunomodulatory activities [15]. CAPE also inhibits enzymatic activities of endogenous and viral proteins [17]–[19] and transcriptional activity of NF-kappaB [14], [20]. In addition, CAPE could suppress HCV replication enhanced by using the NF-kappaB activation activity of morphine [21], although it has been unknown which of moieties including CAPE is responsible for anti-HCV activity. Furthermore, it is not clear whether chemical modification of CAPE could enhance anti-HCV activity or not. In this report, we examined the effect of CAPE derivatives on HCV proliferation to develop more effective and safer anti-HCV compounds.

## Results

### Effect of CAPE on HCV RNA replication in HCV subgenomic replicon cells

CAPE is composed of ester of caffeic acid and phenethyl alcohol (Fig. 1A). We examined the effect of CAPE (compound **1**) on both viral replication and cell growth in the HCV subgenomic replicon cell line Huh7/Rep-Feo. The replicon cell line was treated with various concentrations of compound **1**. The replication level of the HCV RNA was measured as an enzymatic activity of luciferase, which is bicistronically, encoded on the replicon RNA. Compound **1** suppressed HCV RNA replication at concentrations from 1.3 to 40 µM in a dose-dependent manner, but did not affect cell viability (Fig. 1B). HCV NS3, which is a viral protease, was decreased at the protein level by treatment with CAPE in a dose-dependent manner, corresponding to the viral replication, whereas beta-actin was not changed in the replicon cell line (Fig. 1C). Based on the calculation using a dose dependency of CAPE, compound **1** exhibited an EC_50_ value of 9.0 µM and a CC_50_ value of 136.1 µM, giving a selectivity index estimate (SI) of 17.9 (Table 1). These results suggest that treatment with CAPE inhibits HCV replication in HCV subgenomic replicon cells.

**Table 1:** Effect of CAPE (1) and related compounds 2–6 on HCV replication.

Compound (Number)	EC_50_^ a^ (µM)	CC_50_^b^ (µM)	SI ^c^	*C*log *P* ^d^
CAPE (**1**)	9.0±0.7	136.1±1.9	17.9	3.30
caffeic acid (**2**)	36.6±6.7	>320	>8.7	0.98
ferulic acid (**3**)	71.9±5.8	>320	>4.5	1.42
cinnamic acid henethyl ester (**4**)	86.1±6.3	>320	>3.7	4.56
chlorogenic acid (**5**)	103.0±3.4	>320	>3.1	−0.96
rosmarinic acid (**6**)	109.6±1.1	>320	>2.9	1.10

a: Fifty percent effective concentration based on the inhibition of HCV replication.

b: Fifty percent cytotoxicity concentration based on the reduction in cell viability.

c: Selectivity index (CC_50_/EC_50_).

d: Determined with ChemDraw software (Chem Bio Office Ultra, 2008).

### Structure-activity relationship of CAPE analogues

To clarify the structure-activity relationship of CAPE analogues, we examined the effect of hydroxyl groups on the aromatic ring (catechol moiety), the alkenyl moieties on alpha, beta-unsaturated esters, and the ester parts as follows (Figure S1).

We tested whether commercially available CAPE-related compounds **2** to **6** (Fig. S1) affected HCV replication (Table 1). All these compounds showed weaker inhibitory activity than CAPE (**1**), but are not toxic. Compound **2**, which is the acid component of CAPE, showed a slightly lower value of EC_50_ than compound **3**, which is the compound **2** derivative replaced a hydroxyl group with a methoxyl group of catechol moiety, while compound **4**, which is the derivative lacking two hydroxyl groups within catechol moiety, exhibits a higher value of EC_50_ than compounds **1** and **2**. These data suggest that the catechol moiety of CAPE is required for anti-HCV activity. Interestingly, compounds **5** and **6**, which are natural products including polyhydroxylated acid moieties in the ester parts, showed much weaker inhibitions than compound **1** and exhibits low *C*log *P* values. The position of hydroxyl group or/and the structure of the ester part may affect the inhibitory activity and/or hydrophobicity.

**Figure 2:**
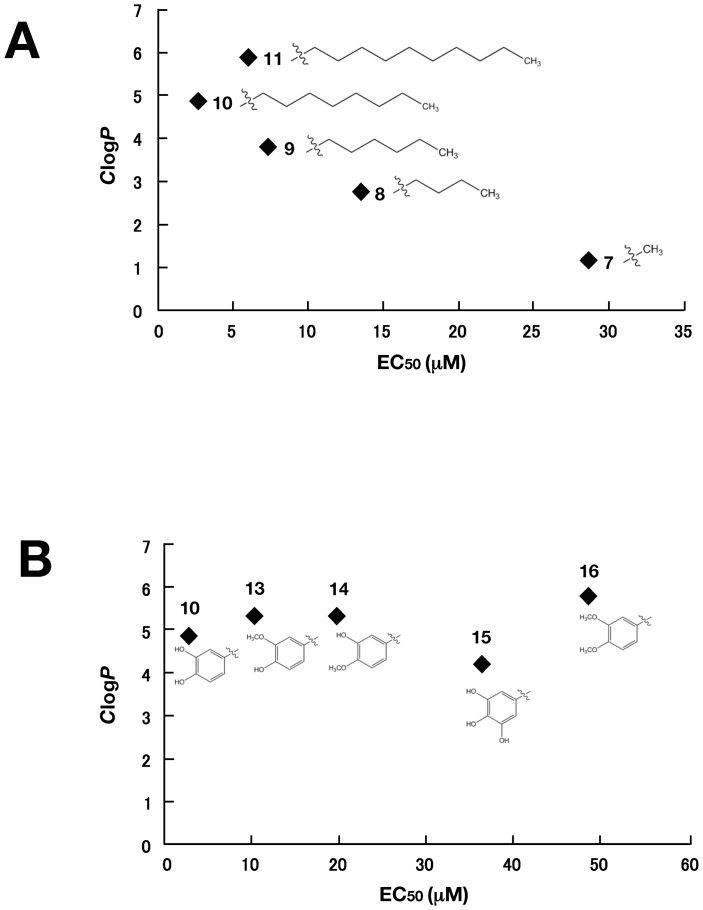
Correlation between the inhibitory effect on HCV replication *and C*log *P* of CAPE analogues. Values of *x*-axis indicate EC_50_ values of CAPE analogues, while values of *y*-axis show *C*log *P* values. (A) Correlation between the inhibitory effect on HCV replication and *C*log *P* of CAPE analogues (Compound 7–11). (B) Correlation between the inhibitory effect on HCV replication and *C*log *P* of CAPE analogues (Compound 10 and 13–16).

We next examined the effects of caffeic acid ester compounds **7** to **11**, which include various lengths of alkyl side chains, on HCV replication (Table 2 and Figure S2). The EC_50_ values decreased in the order methyl ester (compound **7**), n-butyl ester (compound **8**), n-hexyl ester (compound **9**), and n-octyl ester (compound **10**), suggesting that elongation of the n-alkyl side chain increased the inhibitory activity. However, the EC_50_ value of n-dodecyl ester (compound **11**) was higher than that of compound **10**. Thus, n-octyl ester (compound **10**) showed the lowest EC_50_ value and the highest SI among the tested compounds shown in Tables 1 and 2. Compounds **7** to **11** gradually increased own *C*log *P* values, corresponding to length of n-alkyl side chain (Fig. 2A). Compounds **10** and **11** exhibit EC_50_ values of 2.7 and 5.9 µM, respectively, SI values of 29.6 and 9.80, respectively, and *C*log *P* values of 4.90 and 5.96, respectively, suggesting that high hydrophobic property of n-alkyl side chain decreases anti-HCV activity. The appropriate *C*log *P* value of caffeic acid ester containing unsaturated side chain may be around 5.

**Table 2:** Effect of caffeic acid esters 7, 9–14, including 1, on HCV replication.

Compound No.	R	EC_50_^a^ (µM)	CC_50_^b^ (µM)	SI ^c^	*C*log *P* ^d^
**7**	CH_3_	28.6±1.2	122.1±5.0	4.2	1.20
**8**	C_4_H_9_	13.5±2.1	39.0±1.1	2.9	2.79
**9**	C_6_H_13_	7.3±0.2	37.6±1.2	5.1	3.85
**10**	C_8_H_17_	2.7±0.1	71.7±8.5	26.6	4.90
**11**	C_10_H_21_	5.9±0.9	57.9±2.9	9.8	5.96
**1**	(CH_2_)_2_Ph	9.0±0.7	136.1±1.9	17.9	3.30

The basic structure and side moieties are shown in Figure S2.

a: Fifty percent effective concentration based on the inhibition of HCV replication.

b: Fifty percent cytotoxicity concentration based on the reduction in cell viability.

c: Selectivity index (CC_50_/EC_50_).

d: Determined with ChemDraw software (Chem Bio Office Ultra, 2008).

Dihydrocaffeic acid methyl ester (compound **12**) showed less activity than caffeic acid methyl ester (compound **7**) regardless of values of *C*log *P* value and CC_50_, suggesting that the alpha, beta-unsaturated part attached to ester affects the anti-HCV activity level (Table 3 and Figure S3).

**Table 3:** Effect of caffeic acid esters 7and 8 on HCV replication.

Compound No.	EC_50_^a^ (µM)	CC_50_^b^ (µM)	SI ^c^	*C*log *P* ^d^
**7**	28.6±1.2	122.1±5.0	4.2	1.20
**12**	77.0±1.6	140.7±3.4	1.8	1.02

Chemical structures of both compounds are shown in Figure S3

a: Fifty percent effective concentration based on the inhibition of HCV replication.

b: Fifty percent cytotoxicity concentration based on the reduction in cell viability.

c: Selectivity index (CC_50_/EC_50_).

d: Determined with ChemDraw software (Chem Bio Office Ultra, 2008).

We further examined the effect of the hydroxyl groups on the aromatic ring on HCV replication (Table 4 and Figure S4). The EC_50_ values of *O*-methylated caffeic acid n-octyl esters (compounds **13** and **14**) were higher than that of compound **10**. Compounds **15** including 3, 4-di-*O*-methylated caffeic acid n-octyl ester exhibited higher EC_50_ than values of compounds **10**, **13** and **14**. However, addition of a third hydroxyl group to 3, 4, 5-trihydroxy derivative (compound **16**) of compound **10** resulted in a reduction of anti-HCV activity. Furthermore, *C*log *P* values of compound **10**, **13**, **14, 15** and **16** were not correlated with anti-HCV activity (EC_50_ value) (Fig. 2B). These results suggest that the catechol moiety plays an important role in anti-HCV activity, and that the 4-hydroxy moiety is more important for the activity than the 3-hydroxy moiety.

**Table 4:** Effect of octyl esters 10 and 13–16 on HCV replication.

Compound No.	R^1^, R^2^, R^3^	EC_50_^a^ (µM)	CC_50_^b^ (µM)	SI ^c^	*C*log *P* ^d^
**10**	R^1^ = R^2^ = R^3^ = H	2.7±0.1	71.7±8.5	26.6	4.90
**13**	R^1^ = CH_3_, R^2^ = R^3^ = H	10.2±1.1	60.3±1.6	5.9	5.35
**14**	R^1^ = R^3^ = H, R^2^ = CH_3_	19.6±0.8	59.2±1.4	3	5.35
**15**	R^1^ = R^2^ = CH_3_, R^3^ = H	48.5±1.7	212.4±6.9	4.4	5.82
**16**	R^1^ = R^2^ = H, R^3^ = OH	36.3±2.9	59.8±6.9	1.6	4.24

The basic structure and side moieties are shown in Figure S4.

a: Fifty percent effective concentration based on the inhibition of HCV replication.

b: Fifty percent cytotoxicity concentration based on the reduction in cell viability.

c: Selectivity index (CC_50_/EC_50_).

d: Determined with ChemDraw software (Chem Bio Office Ultra, 2008).

Thus, compound **10**, which exhibits the lowest EC_50_ value and the highest SI value, is the most effective compound among CAPE analogues used in this study.

### Effect of CAPE derivatives on virus production

**Figure 3:**
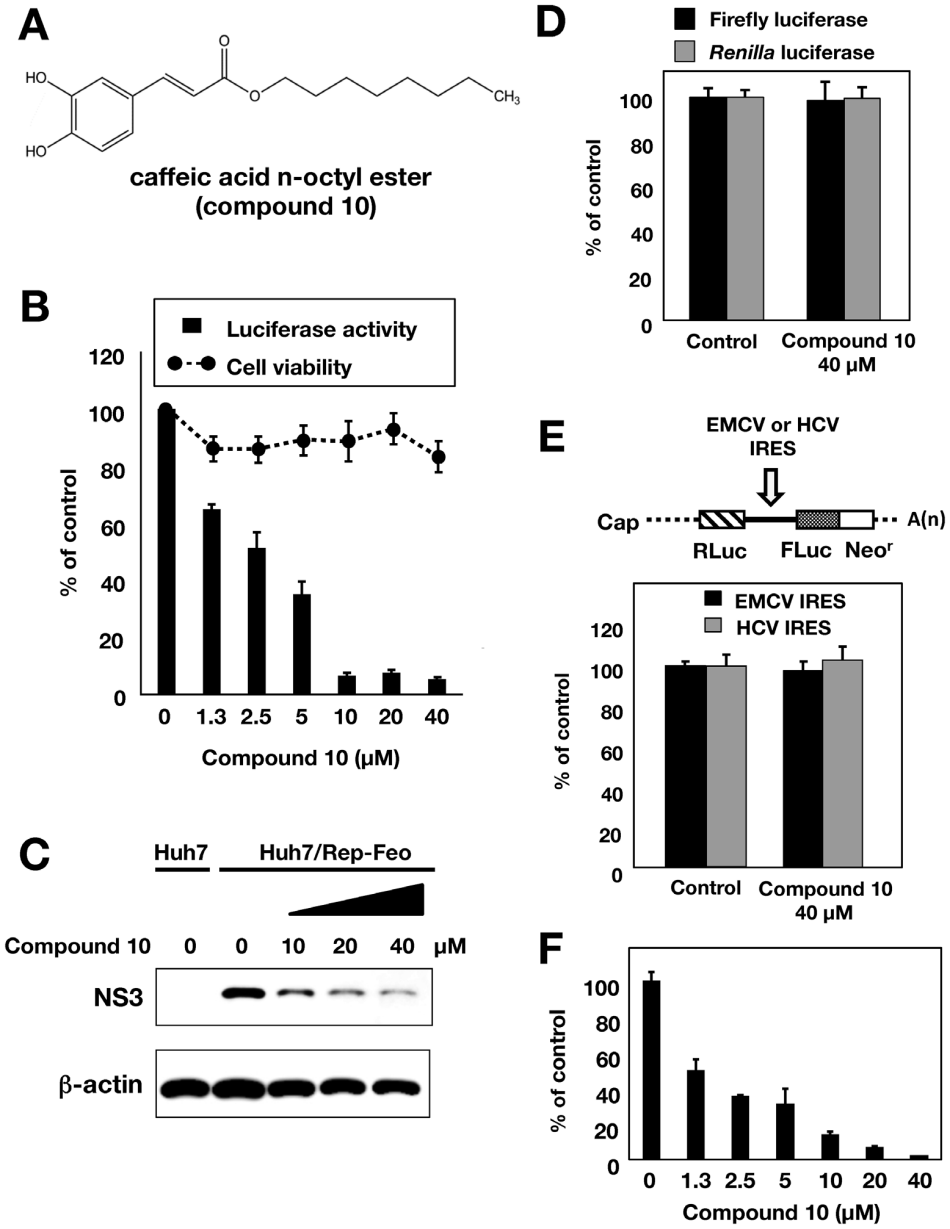
Effect of compound 10 on the viral replication in the replicon cell line and HCVcc. (A) Molecular structure of compound **10**. (B) Huh7/Rep-Feo cells were incubated for 72 h in a medium containing various concentrations of compound **10**. Luciferase and cytotoxicity assays were carried out by the method described in Materials and Methods. Error bars indicate standard deviation. The data represent three independent experiments. (C) Protein extract was prepared from Huh7/Rep-Feo cells treated for 72 h with the indicated concentration of compound **10** and it was then subjected to Western blotting using antibodies to NS3 and beta-actin. (D) Huh7 cell line was transfected with pEF Fluc IN encoding firefly luciferase or pEF Rluc IN encoding *Renilla* luciferase. Both transfected cell lines were incubated with DMSO (Control) or 40 µg/ml compound **10**. Firefly or *Renilla* luciferase activity was measured 72 h post-treatment. Luciferase activity was normalized with protein concentration. Error bars indicate standard deviation. The data were represented from three independent experiments. (E) Schematic structure of RNA transcribed from the plasmids was shown (Top). The bicistronic gene is transcribed under the control of elongation factor 1α (EF1α) promoter. The upstream cistron encoding *Renilla* luciferase (RLuc) is translated by a cap-dependent mechanism. The downstream cistron encodes the fusion protein (Feo), which consists of the firefly luciferase (Fluc) and neomycin phosphotransferase (Neo^r^), and is translated under the control of the EMCV or HCV IRES. Huh7 cell line was transfected with each plasmid and incubated for 72 h post-treatment with DMSO (control) or 40 µg/ml of compound **10**. Firefly and *Renilla* luciferase activities were measured. Relative ratio of Firefly luciferase activity to *Renilla* luciferase activity was represented as percentage of the control condition. Error bars indicate standard deviation. The data were represented from three independent experiments. (F) Huh7 OK1 cell line was infected with HCVcc derived from JFH-1 strain and then treated with several concentrations of compound **10** at 24 h post-infection. The resulting cells were harvested 72 h post-infection. The viral RNA of supernatant was purified and estimated by the method described in Materials and Methods. Error bars indicate standard deviation. The data represent three independent experiments. Treatment with DMSO corresponds to ‘0’.

**Figure 4:**
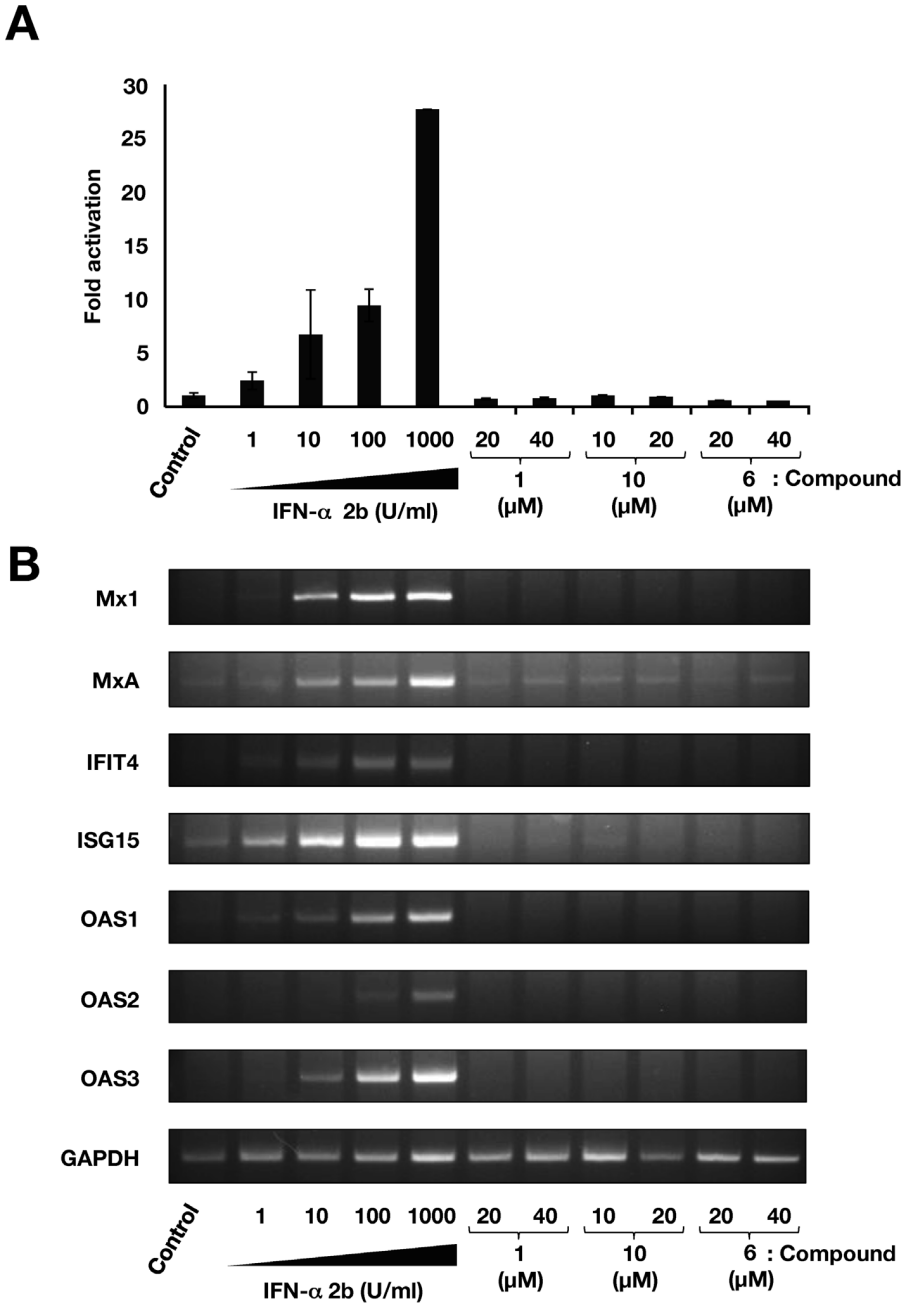
Effect of CAPE derivatives on the interferon-signaling pathway. (A) Plasmids pISRE-TA-Luc and phRG-TK were co-transfect into Huh7 OK1 cells. The transfected cells were cultured with 1, 10, 100, or 1000 U/mL of interferon-alpha 2b, and compounds **1**, **6** and **10**. Treatment with DMSO corresponds to ‘0’. After 48 h of treatment, luciferase activities were measured, and the value were normalized against *Renilla* luciferase activities. Error bars indicate standard deviation. The data represent three independent experiments. (B) Huh7 replicon cell line of genotype 1b was treated with 1, 10, 100, or 1000 U/mL of interferon-alpha 2b, and compounds **1**, **6** and **10** for 48 h. Treatment with DMSO corresponds to the control. The mRNAs of Mx1, MxA, IFIT4, ISG15, OAS1, OAS2, OAS3, and GAPDH as an internal control were detected by RT-PCR.

The structure of compound **10** is shown in Fig. 3A. Treatment with compound **10** reduced HCV replication and NS3 protein in a dose-dependent manner at a higher anti-HCV level than compound **1** (Figs. 3B and C), but not effect enzymatic activities of firefly and *Renilla* luciferases (Fig. 3D) and IRES-dependent translation (Fig. 3E), suggesting that inhibition of HCV replication by compound **10** is not due to offtarget effect. We evaluated the inhibitory effect of compound **10** on three different subgenomic replicon cell lines (1b: N strain, Con1 strain, 2a: JFH-1 strain) and one full genome replicon cell line (1b: O strain). Compound **10** inhibited the viral replication of all replicon cell lines at similar level, and exhibited the lowest EC_50_ value of 1.0 µM and an SI value of 63.1 by using Con1 replicon cells (Table 5). We next examined the effect of compound **10** on virus production by using HCVcc, since subgenomic replicon mimics HCV replication, but not the whole viral cycle. The Huh7 OK1 cell line, which is highly permissive to the HCV JFH1 strain [22], was infected with HCVcc and then treated with compound **10** at 24 h post-infection. The supernatant was harvested 72 h post-infection from the culture supernatant and then the RNA that prepared from the supernatant was estimated by real time qRT-PCR. Figure 3F shows that treatment with compound **10** reduced HCV viral production (EC_50_ = 1.8±0.4 µM) in a similar way to the data obtained by using a replicon cell line. To clarify whether or not compound **10** inhibited HCV replication via interferon-signaling pathway, we analyzed ISRE activity and the expression of interferon stimulated gene (ISG) by using reporter assay and RT-PCR, respectively. The replicon cells were harvested at 48 h post-treatment. There were no significant effects of compound **1**, **6** and **10** on ISRE-promoter activities, while interferon alpha 2b significantly enhanced it as a positive control (Fig.4 A). The data of the RT-PCR analysis showed that the transcriptional expressions of ISGs including Mx1, MxA, IFIT4, ISG15, OAS1, OAS2, and OAS3 were induced with interferon alpha 2b, but not with compound **1**, **6** and **10** (Fig. 4B). These data suggest that the CAPE derivatives have an inhibitory effect on virus production and replication, irrespective of interferon signaling induction.

**Table 5:** Anti-HCV activity of compound 10 in replicon cell lines of genotypes 1b and 2a.

Cell line	Replicon type	Strain (Genotype)	EC_50_^a^ (µM)	CC_50_^b^ (µM)	SI ^c^
Huh7 Rep/Feo-1b	Subgenome	N (1b)	2.7±0.1	71.7±8.5	26.6
Con1 LUN Sb #26	Subgenome	Con1 (1b)	1.0±0.1	63.1±3.1	63.1
Huh7 Rep/Reo-2a	Subgenome	JFH1 (2a)	1.0±0.3	60.0±2.3	60.0
OR6	Full genome	O (1b)	1.5±0.4	61.7±0.6	41.1

a: Fifty percent effective concentration based on the inhibition of HCV replication.

b: Fifty percent cytotoxicity concentration based on the inhibition of HCV replication.

c: Selectivity Index (CC_50_/EC_50_).

### Synergistic effect of caffeic acid n-octyl ester on interferon and direct-acting antiviral agents

**Figure 5:**
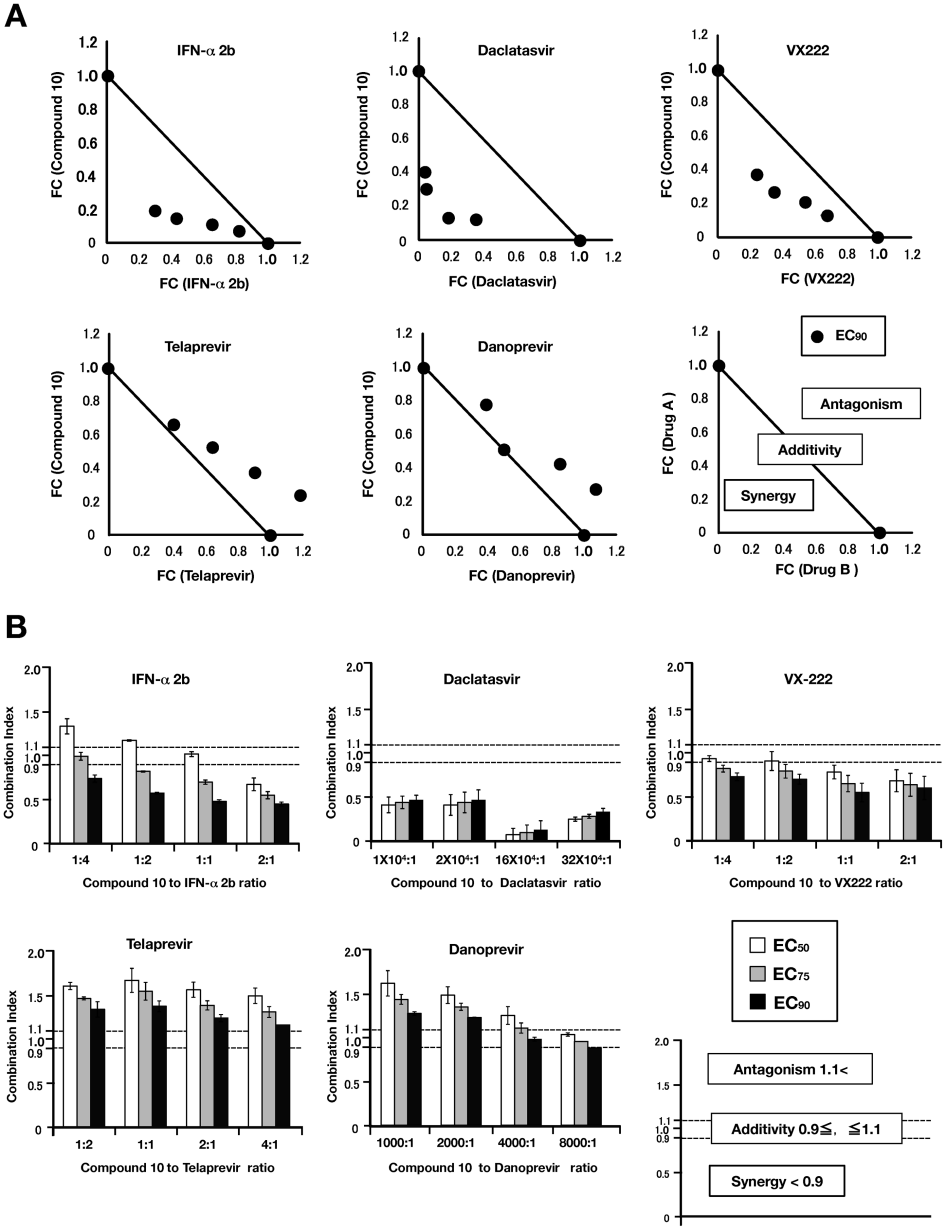
Synergistic effect analyses for the combination of compound 10 with IFN-α 2b, daclatasvir, VX-222, telaprevir, or danoprevir. The Huh7 cell line, including the subgenomic replicon RNA of genotype 1b strain Con1, was treated for 72h with combinations of compound 10 and IFN-α 2b, daclatasvir, VX-222, telaprevir, or danoprevir. Luciferase assay were carried out as described in Materials and Methods. (A) The calculated EC_90_ values for combination were plotted as the fractional concentration (FC) of compound 10 and one of IFN-α 2b, daclatasvir, VX-222, telaprevir, and danoprevir on the *x* and *y* axes, respectively. Synergy, antagonism and additivity are indicated in a representative graph as a right end of lower graphs and are described in Materials and Methods. (B) Combination indexes of compound 10 with IFN-α 2b, daclatasvir, VX-222, telaprevir, or danoprevir at the EC50, EC75, and EC90 values were measured at various drug rations. Synergy, antagonism and additivity are indicated in a representative graph as a right end of lower graphs and are described in Materials and Methods.

To estimate the effects of drug combinations on anti-HCV activity, we examined the antiviral activity of compound **10** in combination with IFN-α 2b, telaprevir (NS3 protease inhibitor), danoprevir (NS3 protease inhibitor), daclatasvir (NS5A inhibitor) or VX-222 (NS5B polymerase inhibitor). Con1 LUN Sb #26 replicon cells were treated with compound **10** in combination with each anti-HCV agent at various concentration rations for 72 h. The effect of each drug combination on HCV replication was analyzed by using CalcuSyn. An explanatory diagram of isobologram was shown at a right end of lower panels of Fig. 5A as described in Materials and Methods. As shown in the resulting isobologram, all plots of the calculated EC_90_ values of compound **10** with IFN-alpha 2b, daclatasvir, or VX-222 are located under the additive line, while the plots of compound **10** with telaprevir, or danoprevir are located above the additive line and closed to the additive line (Fig. 5A). Additionally, we determined the degree of inhibition for each drug combination was analyzed as the combination index (CI) calculation at 50, 75 and 90% of effective concentrations by using CalcuSyn. An explanatory diagram was shown at a right end of lower panels of Fig. 5B as described in Materials and Methods. On the basis of the CalcuSyn analysis, the combination of compound **10** with daclatasvir exhibited strong synergistic effect on inhibition of HCV replication in the replicon cells (Fig. 5B, upper middle). The combination of compound **10** with VX-222 exhibited an additive to synergistic effect, suggesting that it trends toward synergistic (Fig. 5B, upper right), and with IFN-alpha 2b exhibited an antagonistic to synergistic effect, suggesting that it trends toward synergistic (Fig. 5B, upper left). In contrast, the combination of compound **10** with telaprevir resulted in antagonistic effect (Fig. 5B, lower left), and with danoprevir resulted in an antagonistic to additive effect, suggesting it trends toward antagonistic (Fig. 5B, lower middle). These calculated data of combination tests suggest that daclatasvir, IFN-alpha 2b, and VX-222 synergistically, but telaprevir and danoprevir antagonistically, inhibit HCV replication in combination with compound **10**.

## Discussion

CAPE is an active component of propolis, which possesses broad-spectrum biological activities [14]–[19]. In this study, CAPE suppressed HCV RNA replication in a dose-dependent manner (Fig. 1A and B). Treatment with CAPE inhibited HCV replication with an EC_50_ of 9.0 µM and an SI of 17.9 in Huh7/Rep-Feo cells (Table 1). The treatment of the replicon cell line with CAPE did not induce expression of the IFN-inducible gene (Fig. 4), suggesting that the inhibition of HCV replication by CAPE is independent of the IFN signaling pathway.

We also examined the effect of CAPE derivatives on HCV replication. Our data suggest that the n-alkyl side chain and catechol moiety of the CAPE derivative are critical in its anti-HCV activity (Tables 2 and 3). The EC_50_ value of the derivative decreased dependently on the length of the n-alkyl side chain until reaching octyl ester length (Table 2), while longer chains than octyl ester of a derivative led to an increase in the EC_50_ value and *C*log *P* value. Compound **10**, Caffeic acid n-octyl ester, exhibited the highest anti-HCV activity among the tested compounds with an EC_50_ value of 2.7 µM and an SI value of 26.6. Cyclosporine A and its analogues could suppress the viral replication of genotype 1b at a higher level than that of genotype 2a [23]. Interestingly, compound **10** could inhibit HCV replication of genotype 1b and 2a at a similar level, irrespective of expression of the interferon-inducible gene (Fig. 4). CAPE and its derivatives may therefore possess a mechanism different from cyclosporine A and its analogues with respect to anti-HCV activity.

CAPE has been reported to be an inhibitor of NF-kappaB [14], [20]. Lee et al. reported that the catechol moiety in CAPE was important for inhibition of NF-kappaB activation [24]. The data shown in Table 3 suggest that the catechol moiety in CAPE is critical to the anti-HCV activity of compound **10**. Previous studies have implicated the inhibition of NF-kappaB in anti-HCV activity. Treatment with an extract prepared from *Acacia confusa* [25] or San-Huang-Xie-Xin-Tang [26] could suppress HCV replication and inhibit NF-kappaB activation. However, Chen et al. reported that curcumin-mediated inhibition of NF-kappaB did not contribute to anti-HCV activity [11]. Furthermore, treatment with *N′*-(Morpholine-4carbonyloxy)-2(naphthalene-1-yl) acetimidamide could activate NF-kappaB and downstream gene expression in the same Huh7/Rep-Feo replicon cell line as the cell line used in this study and exhibited potent inhibition of HCV replication without interferon signaling [27]. These reports support the notion that CAPE derivatives do not mainly target NF-kappaB activity as part of their anti-HCV activity.

Several host proteins have been reported to regulate function of NS5A, leading to supporting HCV replication (review in [2], [28] ). Daclatasvir exhibited potent synergistic effect on anti-HCV activity in combination of compound **10** (Fig. 5). Anti-HCV activity of compound **10** might associate with intrinsic functions of host factors that interact with NS5A. NS3 protease inhibitors exhibited antagonistic effect in combination of compound **10** (Fig. 5). The inhibitory effect of compound **10** might be mediated by the activation of an unknown endogenous protease that is nonspecifically suppressed by NS3 protease inhibitors. Further study to clarify the mechanism by which compound **10** suppresses HCV replication might contribute to identification of a novel host factor as a drug target for development of the effective compound supporting an effect of other anti-HCV drugs.

In conclusion, we showed that CAPE and its analogue possess a significant inhibitory effect against HCV replication. The length of n-alkyl side chains and the catechol moiety of CAPE are critical to its inhibitory activity against HCV replication. The most effective derivative among the tested compounds was caffeic acid n-octyl ester, which exhibited an EC_50_ value of 1 µM and an SI value of 63.1 in the replicon cell line of genotype 1b strain Con1. Treatment with caffeic acid n-octyl ester reduced the viral replication of genotype 1b and 2a at a similar level and inhibited viral production of HCVcc. Treatment with caffeic acid n-octyl ester could synergistically enhance the anti-HCV activities of IFN-α 2b, daclatasvir, and VX-222, but neither telaprevir nor danoprevir. Further investigation to clarify the mechanism of anti-HCV activity and further modification of the compound to improve anti-HCV activity will lead to novel therapeutic strategies to treat chronic hepatitis C virus infection.

## Materials and Methods

### Compounds

Boldface numbers in this text indicate the compound numbers shown in Tables. All chemical structures of compounds used in this study are shown in figure S1. CAPE (**1**), caffeic acid (**2**), ferulic acid (**3**), and chlorogenic acid (**5**) and were purchased from Sigma-Aldrich (St. Louis, MO, USA). Cinnamic acid phenethyl ester (**4**) was from Tokyo Chemical Industry (Tokyo, Japan). Rosmarinic acid (**6**) was from Wako Pure Chemical (Tokyo, Japan). Caffeic acid n-octyl ester (n-octyl caffeate) (**10**), 3-O-methylcaffeic acid n-octyl ester (n-octyl-3-methylcaffeate) (**13**), 4-O-methylcaffeic acid n-octyl ester (n-octyl-3-methylcaffeate) (**14**), and 3, 4-O-dimethylcaffeic acid n-octyl ester (n-octyl-3, 4-methylcaffeate) (**15**) were from LKT Laboratories (St. Paul, MN, USA).

Caffeic acid esters **7**, **8**, **9**, and **11** were synthesized by preparing caffeic acid chloride followed by treatment with corresponding alcohols [29]. Dihydrocaffeic acid ester **12** was prepared by hydrogenation of **7**. Compound **16** is a newly synthesized ester. Spectroscopic data of known esters **7–9**, and **11** prepared here were identical to those reported [30]-[32] Interferon alfa-2b (IFN-α 2b) was obtained from MSD (Tokyo, Japan). Telaprevir and daclatasvir were purchased from Selleckchem (Houston, TX, USA). Danoprevir and VX-222 were from AdooQ BioScience (Irvine, CA, USA).

### Chemistry of 3,4,5-Trihydroxycinnamic acid n-octyl ester

3,4,5-Trihydroxycinnamic acid n-octyl ester (**16**) was prepared by condensation of corresponding benzaldehydes with malonic acid n-octyl monoester [33]. A solution of malonic acid n-octyl monoester (432 mg, 2 mmol), 3,4,5-trihydroxybenzaldehyde (462 mg, 3 mmol) and piperidine (0.2 mL) in pyridine (2 mL) was heated at 70°C for 1 h. The reaction mixture was concentrated under a vacuum to give a residue, which was dissolved in CHCl_3_-IPA (3∶1, v/v) and then washed with 10% HCl and water. The organic layer was dried over Na_2_SO_4_ and evaporated to give a residue, which was purified by silica gel column chromatography using AcOEt-hexane (1∶1, v/v) as eluent to give the corresponding n-octyl ester (85 mg, 13.8%) as a pale powder. FT-IR νmax (KBr): 3389, 3239, 2923, 1675, 1627, 1606 cm^−1^. ^1^H NMR (400MHz, CD_3_OD) δ: 0.86 (3H, t, *J* = 7.2 Hz), 1.20–1.40 (10H, m), 1.65 (2H, quintet, *J* = 6.4 Hz), 4.11, (2H, t, *J* = 6.4 Hz), 6.16 (2H, d, *J* = 15.6 Hz), 6.55 (2H, s), 7.40 (2H, d, *J* = 15.6 Hz). 13C NMR (100 Hz, CD_3_OD) δ: 14.4, 23.7, 27.1, 29.8, 30.3, 30.4, 32.9, 65.6, 108.5, 115.3, 126.6, 137.5, 147.1, 169.4. CI MS *m/z*: 309 (M^+^+H). High-resolution CI MS calcd. for C_17_H_25_O_5_ (M^+^+H) for 309.1702. Found: 309.1686.

### Replicon cell lines and virus infection

The Huh7/Rep-Feo cell line, which harbors the subgenomic replicon RNA composed of HCV IRES, the gene of the fusion protein consisting of neomycin phosphotransferase and firefly luciferase, EMCV IRES and a nonstructural gene of genotype 1b strain N in order in Huh7 cell line, was previously established [34]. Thus, the luciferase activity corresponds to the level of HCV RNA replication. The cell line was maintained in Dulbecco's modified Eagle medium containing 10% fetal calf serum and 0.5 mg/mL G418 and cultured in absence of G418 when they were treated with compounds. The Lunet/Con1LUN Sb #26 cell line, which harbors the subgenomic replicon RNA of the Con1 strain (genotype 1b), was described previously [35]. The OR6 cell line, which harbors the full genomic replicon RNA of the O strain (genotype 1b), was described previously [36]. The HCV replicon cell line derived from the genotype 2a strain JFH1 was described previously [37]. The viral RNA derived from the plasmid pJFH1 was transcribed and introduced into Huh7OK1 cells according to the method of Wakita et al. [38]. The virus was amplified by the several times passages. The cells were infected with the virus at a multiplicity of infection (moi) of 1 and then treated with each compound at 24 h post-infection. The culture supernatants were harvested 72 h post-treatment to estimate the viral RNA as described below.

### Determination of luciferase activity in HCV replicon cells

The replicon cells were seeded at 2×10^4^ cells per well in a 48-well plate 24 h before treatment. Compounds were added to the culture medium to give various concentrations. The resulting cells were harvested 72 h post-treatment and lysed with cell culture lysis reagent (Promega, Madison, WI). The luciferase activity of each cell lysate was estimated using a luciferase assay system (Promega). The resulting luminescence was detected by a Luminescencer-JNR AB-2100 (ATTO, Tokyo, Japan).

### Determination of Cytotoxicity in HCV replicon cells

The replicon cells were seeded at a density of 1×10^4^ cells per well in a 96-well plate and then incubated at 37°C for 24 h. Compounds were added to the culture medium to give various concentrations and were then harvested 72 h post-treatment. Cell viability was measured using a dimethylthiazol carboxymethoxy-phenylsulfophenyl tetrazolium (MTS) assay with a CellTiter 96 aqueous one-solution cell proliferation assay kit (Promega).

### Western Blotting

Western blotting was carried out by the method described previously [39]. The antibodies to NS3 (clone 8G-2, mousemonoclonal, Abcam, Cambridge, UK), and beta-actin were purchased from Cell Signaling Technology (rabbit polyclonal, Danvers, MA, USA) and were used as the primary antibodies in this study.

### RNA analysis

Total RNAs were prepared from cells by using the RNAqueous-4PCR kit (Life Technologies, Carlsbad, CA). Viral RNA were prepared from culture supernatants by using the QIAamp Viral RNA mini kit (QIAGEN, Hilden, Germany). The viral RNA genome was estimated by the qRT-PCR method described previously [40]. RT-PCR was carried out by the method described previously [41] which was slightly modified at the PCR step. The PCR samples were incubated once for 10 min at 95°C for an initial activation step of the AmpliTaq Gold DNA Polymerase (Life Technologies), and then subjected to an amplification step of 30 repeats of the cycle consisting of three segments as follow: 0.5 min at 95°C, 1 min at 55°C and 1 min at 72°C. The primers used in this study were as follows: Mx1: 5′-AGCCACTGGACTGACGACTT-3′ and 5′-GAGGGCTGAAAATCCCTTTC-3′;

MxA: 5′-GTCAGGAGTTGCCCTTCCCA-3′ and 5′-ATTCCCATTCCTTCCCCGG-3′;

IFIT4: 5′-CCCTTCAGGCATAGGCAGTA-3′ and 5′-CTCCTACCCGTCACAACCAC -3′; ISG15: 5′-CGCAGATCACCCAGAAGAT-3′ and 5′-GCCCTTGTTATTCCTCACCA-3′;

OAS1: 5′-CAAGCTCAAGAGCCTCATCC-3′ and 5′-TGGGCTGTGTTGAAATGTGT-3′;

OAS2: 5′-ACAGCTGAAAGCCTTTTGGA-3′ and 5′-GCATTAAAGGCAGGAAGCAC-3′;

OAS3: 5′-CACTGACATCCCAGACGATG-3′ and 5′-GATCAGGCTCTTCAGCTTGGC-3′;

GAPDH: 5′-GAAGGTGAAGGTCGGAGTC and 5′- GAAGATGGTGATGGGATTTC-3′

### Effects on activities of internal ribosome entry site (IRES) and luciferases

Huh7 OK1 cells were transfected with pEF.Rluc.HCV.IRES.Feo or pEF.Rluc.EMCV.IRES.Feo [39]. These transfected cells were seeded at 2×10^4^ cells per well in a 48-well plate 24 h before treatment, treated with DMSO or compound **10**, and then harvested at 72 h post-treatment. The firefly luciferase activities were measured with a luciferase assay system (Promega). The total protein concentration was measured using the BCA Protein Assay Reagent Kit (Thermo Scientific, Rockford, IL, USA) to normalize luciferase activity. To evaluate the interferon response, Huh7OK1 cells were seeded on a 48 well plate at a density of 2×10^4^ cells per well, and transfected with pISRE-TA-Luc (Takara bio, Shiga, Japan) and phRG-TK (Promega). These transfected cells were incubated in the presence of compounds, IFN-α 2b, or DMSO, and then harvested at 48 h post-treatment. The firefly luciferase and *Renilla* luciferase activities were quantified by using Dual luciferase reporter assay system (Promega).

### Prediction of Clog*P* for compounds

The *C*log*P* value deduced from chemical structure roughly corresponds to a value of hydrophobicity. The *C*log*P* values of compounds used in this study were calculated using the computer software Chem Bio Office Ultra 2008 (PerkinElmer, Cambridge, MA, USA).

### Synergistic effect of caffeic acid n-octyl ester on anti-HCV activities of other drugs

The effects of drug-drug combinations were evaluated by using the Con1 LUN Sb #26 replicon cells, and were analyzed by using the computer software CalcuSyn (Biosoft, Cambridge, United Kingdom). Dose inhibition curves of two different drugs were plotted with each other. In each drug combination, EC_90_ values of several combinations of two different drugs were plotted as the fractional concentration (FC) of both drugs on the *x* and *y*-axes. Additivity indicates the line linked between 1.0 FC value points of both drugs in the absence of each other. Synergy and antagonism are indicated by values plotted under and above, respectively, an additivity line. The explanatory diagram of isobologram is shown in a right end of lower panels of Figure 5A. Combination indexes (CIs) were calculated at the EC_50_, EC_75_, and EC_90_ by using CalcuSyn. A CI value of less than 0.9 indicates synergy. A CI value ranging from 0.9 to 1.1 indicates additivity. A CI value of more than 1.1 indicates antagonism. The explanatory diagram was shown in a right end of lower panels of Figure 5B.

## Supporting Information

Figure S1 **Molecular structure of CAPE and commercial CAPE-related compounds.** CAPE structure is divided into three parts: (I) the catechol moiety, (II) the alkenyl moiety on alpha, beta -unsaturated ester, and (III) the ester part. Molecular structures of CAPE and its commercial derivatives are shown.
(TIF)

Figure S2 **The basic structure and side moieties of compounds shown in** **Table 2** **.** Each compound structure is represented on the basis of the basic structure (top).
(TIF)

Figure S3 **The molecular structures of compounds 7 and 12, which are shown in** **Table 3** **.** Both compounds are different in alpha, beta-unsaturated or saturated part attached to ester.
(TIF)

Figure S4 **The basic structure and side moieties of compounds shown in** **Table 4** **.** Each compound structure is represented on the basis of the basic structure (top).
(TIF)
