# Supplementary figures and images for: Inhibitory Effects of Caffeic Acid Phenethyl Ester Derivatives on Replication of Hepatitis C Virus
Source: PLoS One. 2013 Dec 17;8(12):e82299. doi: 10.1371/journal.pone.0082299 (PMC3866116; doi:10.1371/journal.pone.0082299)

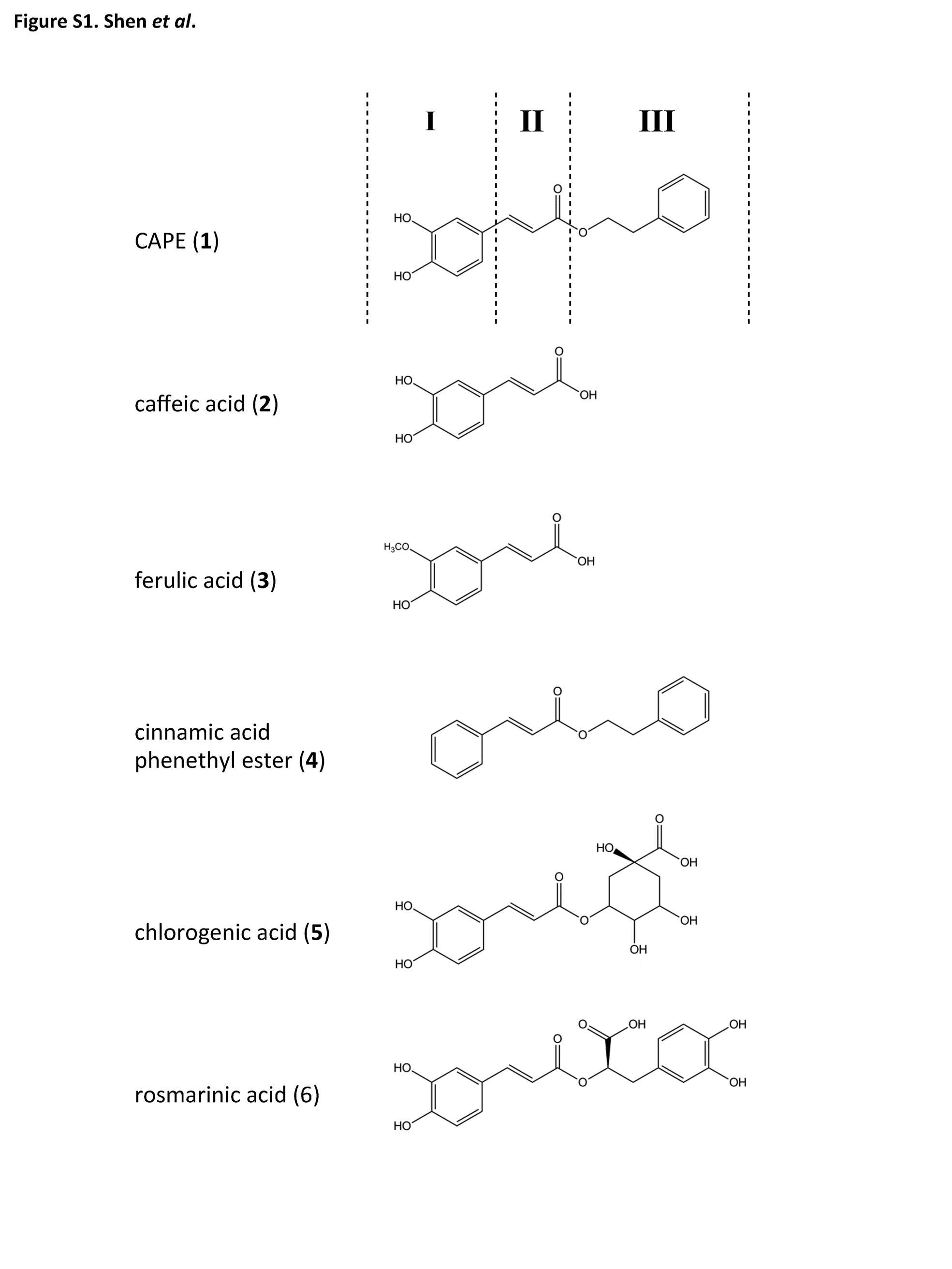

Supplement: Figure S1 — Molecular structure of CAPE and commercial CAPE-related compounds. CAPE structure is divided into three parts: (I) the catechol moiety, (II) the alkenyl moiety on alpha, beta -unsaturated ester, and (III) the ester part. Molecular structures of CAPE and its commercial derivatives are shown. (TIF) [file pone.0082299.s001.tif]

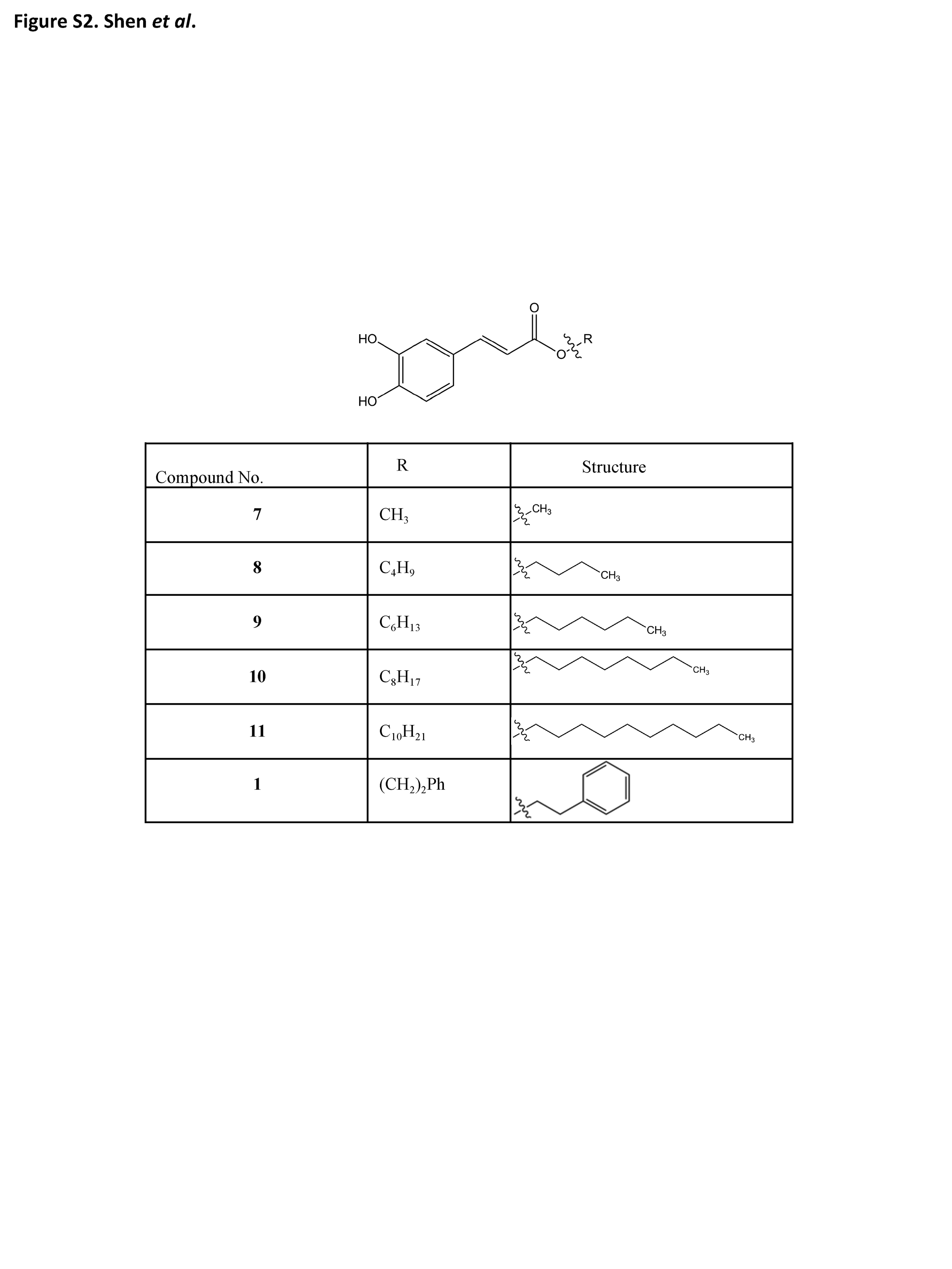

Supplement: Figure S2 — The basic structure and side moieties of compounds shown in Table 2 . Each compound structure is represented on the basis of the basic structure (top). (TIF) [file pone.0082299.s002.tif]

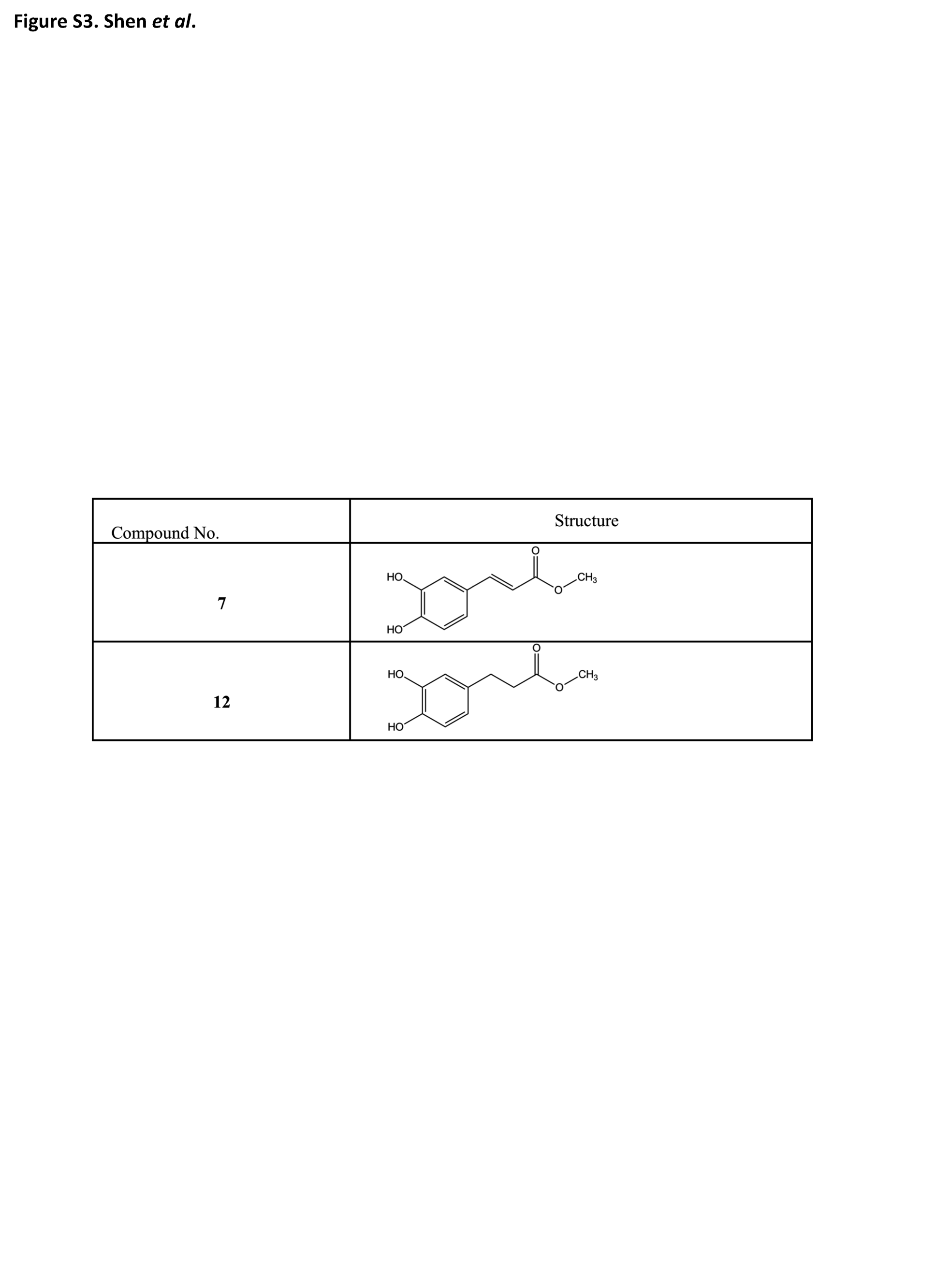

Supplement: Figure S3 — The molecular structures of compounds 7 and 12, which are shown in Table 3 . Both compounds are different in alpha, beta-unsaturated or saturated part attached to ester. (TIF) [file pone.0082299.s003.tif]

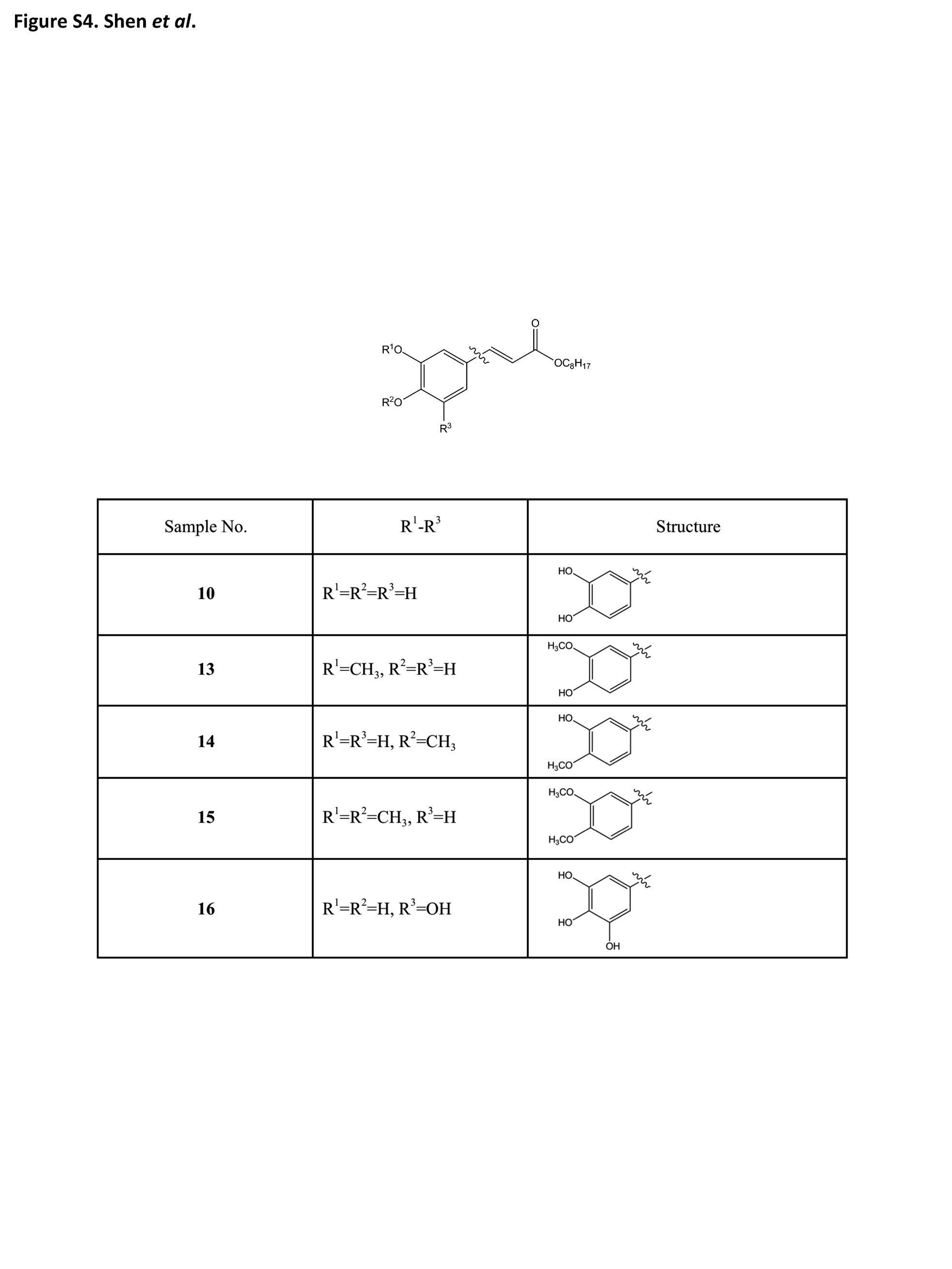

Supplement: Figure S4 — The basic structure and side moieties of compounds shown in Table 4 . Each compound structure is represented on the basis of the basic structure (top). (TIF) [file pone.0082299.s004.tif]
